# Implementing Evidence-Based Creative Arts Therapies in Facilities catering for Older Adults

**DOI:** 10.1093/geroni/igaf122.1775

**Published:** 2025-12-31

**Authors:** Daphna Kalir, Shoshi Keisari, Hod Orkibi, Reem Nashef-Hamuda, Neta Singer

**Affiliations:** University of Haifa, Tel Aviv, Tel Aviv, Israel; University of Haifa, Haifa, HaZafon, Israel; University of Haifa, Haifa, HaZafon, Israel; University of Haifa, Tel Aviv University, Haifa, HaZafon, Israel; University of Haifa, Haifa, HaZafon, Israel

## Abstract

Implementing Evidence-Based Creative Arts Therapies in Facilities catering for Older Adults Daphna M. Kalir, Shoshi Keisari, Hod Orkibi, Rim Abu-Nashef, Neta Singer Abstract Creative Arts Therapies (CATs) utilize various art forms to foster psychological growth, particularly among older adults. The current study explored the implementation patterns of CATs in facilities that cater to older adults’ needs in Israel, and especially the barriers and facilitating factors of the implementation processes. Our research consisted of two studies: a qualitative study aimed to identify barriers and facilitators to CATs implementation, and a quantitative study that included a survey to characterize CATs implementation in Israel. The qualitative sample (n = 18) included managers, social workers, CATs therapists, and older adult clients in community settings. The quantitative study distributed a structured survey to 62 CAT therapists treating older adults, analyzing therapist demographics, practice settings, and key variables. Subgroup comparisons highlighted differences in training, supervision, and work environments, offering a comprehensive view of CATs implementation. Barriers to CATs implementation included a lack of legally established standards, insufficient funding, preconceptions about CATs and mental health among older adults, and decision makers, and limited awareness of the field. Facilitating factors included prior familiarity of managers and policymakers with CATs, recognition of their effectiveness, therapists’ strong motivation, flexible intervention strategies, and implementation frameworks. Findings underscore the need to raise awareness of CATs’ benefits and ensure accessible mental health care for community-dwelling older adults. Addressing misconceptions about CATs and mental health treatment is essential for improving attitudes among older adults and policymakers.

